# On the Effects of Mercury on the Young Subject

**Published:** 1847-02

**Authors:** 


*On the Effects of Mercury on the Young Subject.* By JOHN B. BECK, M. D., *Prof. of Materia Medica and Medical Jurisprudence, in the College of Physicians and Surgeons, of New-York.*

In some previous papers,* I endeavored to point out the peculiarities attending the operation of Opium and Emetics, on the infant subject, as distinguished from the effects of these agents on the adult. I now propose to make some remarks on another article of even still greater importance, and that is *Mercury*. That Mercury is an agent of immense power, either for good or evil, upon the human constitution, cannot be questioned. While in many cases it is the means of saving life, in not a few it unquestionably destroys it. If this be so, it becomes a question of the deepest practical interest, to determine whether its action is modified in any way by the age of the patient, and particularly so, when it is recollected that it is given by too many physicians, even more freely, and may I not add indiscriminately, to the young subject than to the adult.

*The first and most striking peculiarity attending the action of mercury, is that in young subjects, it does not produce salivation so readily as it does in adults.* Indeed under a certain age, it appears to be exceedingly difficult to excite salivation at all in them. On this point, besides our own experience, we have abundance of testimony. Dr. Clark says "under various circumstances he has prescribed mercury, in very large quantities, and in a great number of cases; and he never produced salivation, except in three instances, in any child under three years of age."† Dr. Warren, of Boston, observes, "that he has never known an infant to be salivated, notwithstanding he has given in some cases, large quantities with this view."‡ Mr. Colles, of Dublin, says, "no man in

---

* New-York Journal of Medicine and the Collateral Sciences. Vol. 2, p. 1. Vol. 7, p 153.

† Commentaries on some of the more important diseases of Children. By John Clarke, M. D. p. 182.

‡ View of the Mercurial Practice in Febrile Diseases. By John Warren, M. D. p. 146.

the present day requires to be told that mercury never does produce ptyalism, or swelling and ulceration of the gums in infants."§ Drs. Evanson and Maunsell speak still more strongly. They say, "mercury does not seem capable of salivating an infant. We have never seen it do so, nor are we aware of any such case being on record." "We have never succeeded in salivating a child under three years of age."||

The same general fact seems to be applicable to the external use of mercury. Dr. Percival, of Manchester, remarks, that he, "repeatedly observed that very large quantities of the Unguentum Cœruleum may be used in infancy and childhood, without affecting the gums, notwithstanding the predisposition to a flux of saliva, at a period of life incident to dentition."¶

That salivation does not take place so readily in the infant as in the adult, would seem then to be well establised. That it never can or does take place, as might be inferred from some of the preceding quotations, is by no means, however, true; and the statement, if implicitly relied on, is calculated to be the cause of much mischief. That very young subjects do sometimes become salivated, is unquestionable. One case, and only one, however, has occurred in my experience, in which a child of two years of age was salivated, and that by a very moderate quantity of calomel, viz., five grains, given in three portions, at intervals, within the space of about twelve hours. In about two days after, the gums became inflamed, the tongue swelled, several ulcers appeared in the mouth, and the flow of saliva was free; after continuing about three days in the same state, it gradually yielded, and disappeared without any further inconvenience. In this case every thing seemed favorable to the development of mercurial action. The child had been laboring under hooping cough for several weeks, and was a good deal reduced. It vomited freely with every paroxysm of coughing, and this no doubt aided in bringing on salivation, in a constitution peculiarly sensitive and evidently scrofulous. Nor is this a solitary case. Dr. Clarke, already quoted, admits that in three cases salivation was produced in children under three years of age. And similar cases have been observed by others. Dr. Blackall relates the case of a child, two years of age, who was salivated in consequence of taking two grains of calomel for several successive nights. The child was a poor scrofulous subject, and it sunk under the effects of the mercury.

This, then, is a remarkable peculiarity in the action of this agent upon the infant subject, and the observation of it has doubtless led to the belief, too prevalent among some physicians, that it may be given to them to almost any extent with perfect impunity; an error,

---

§ Practical Observations on the Venereal Disease and on the use of Mercury By Abraham Colles, M. D. p. 171. American edition.

|| Treatise on the Management and Diseases of Children. p. 68.

¶ Essays, Medical anp Philosophical. By Thomas Percival, M. D. vol. 2, p. 318.

which, if not in its immediate, yet certainly in its remote effects, has been the prolific source of more mischief, probably, than any of us are aware of.

*Although mercury so seldom salivates infants, yet, notwithstanding this, it cannot be doubted that it affects the system profoundly, and even more so proportionally than it does the adult.* That it should do so appears perfectly natural, when we reflect upon the mode of its operation on the human system. On this subject, I am aware that a great difference of opinion exists. By some, mercury is looked upon as a stimulant; while others view it as a sedative. A familiar acquaintance with its effects, however, will show, I think, that it may be the one or the other, according to circumstances—according to the dose in which it is given—the length of time it is continued, and more especially, the condition of the system at the time of using it. A single large dose of calomel will cause nausea and relaxation, and sometimes unpleasant prostration, while if it be given in smaller doses and repeated frequently, it will occasion irritation of the intestines, and general disturbance of the vascular and nervous systems. In the former case acting as a profound sedative, and in the latter as a stimulant, or rather irritant. That calomel given in large doses operates as a sedative, seems to be proved, not merely by the nausea and prostration which it frequently produces, but by other considerations. In dysentery, for example, in the adult, a dose of 20 grains of calomel will sometimes allay pain and irritation, with as much certainty as a dose of opium. For the purpose of testing the effects of calomel, some interesting experiments were made by Mr. Annesley, which would seem still further to show, that in large doses the action of this agent upon the mucous membrane of the stomach and intestines, is that of a sedative. He took three healthy dogs, and gave to one, ʒj. of calomel, to a second, ʒij., to a third, ʒiij. After this they were tied up in a room.

"The dog which took ʒi. did not appear to feel any kind of sickness, till six or seven hours afterwards, when he vomited a little. He was lively the whole time, and ate his food well; had been purged two or three times; dejections of a black gray color.

The dog which took ʒij. was likewise lively, and ate his food well, vomited two or three times, and was purged more than the other; he passed tape worms, and the dejection were black.

The dog which took ʒiij. was heavy, and apparently uncomfortable the whole day, and did not vomit at all; he was purged, and passed a very long tape worm; dejections also black."

Twenty-four hours after they had taken the calomel, the dogs were all hung, and in five minutes after they were dead, they were examined, and the vascularity of the stomach was found to be in the inverse ratio of the calomel they had taken; i. e. in the dog which had taken ʒiij., the vascularity was the least, and so on.

For the purpose of comparing this with the condition of the stomach of a dog which had taken no calomel at all, an examination of another dog was made; and here the stomach was found to be *more vascular* than in any of the others. From these experiments, Mr. Annesley drew the conclusion, that "the natural and healthy state of the stomach and intestinal canal is that of high vascularity, and that the operation of calomel in large doses, is directly the reverse of inflammatory." *

The foregoing considerations would seem to show that calomel in full doses is a local sedative, and in its general effects, is debilitating to the system at large. Hence its great utility and value as a remedy in many inflammatory diseases.

When, on the other hand, it is given in small and repeated doses, it acts not unfrequently as a local, as well as a general irritant, producing immoderate action of the bowels, and general irritation of the nervous and vascular systems. Now, these, we know, are the effects observed continually in the adult, and is but reasonable to suppose that all of them must, as a matter of course, be aggravated in the more delicate and sensitive system of the infant.

What shows incontestibly that the action of mercury is actually more energetic on the infant than the adult, is the fact, that when salivation does take place in the former, as it sometimes does, its effects are more disastrous. Sloughing of the gums and cheeks, general prostration and death are by no means uncommon occurrences. On this subject, Dr. Blackall justly remarks, "a general opinion prevails, that the constitution of young subjects resist mercury. Its entrance into the system they certainly do resist, more than we could expect; but they are greatly overcome by salivations, and the possible occurrence of such accidents may well set us constantly on our guard." † Dr. Ryan, too, says, "Ptyalism of infants is often followed by sloughing of the gums and cheeks; and this I have known to occur after the use of it in Hydrocephalus." ‡

Besides being more energetic in its action on the infant, mercury is also more uncertain. This must necessarily be the case, and for the same reasons that every other active agent is so. In the adult we know that mercury varies in its effects, according to the condition of the system, and the peculiarities of the patient's constitution. Thus some persons are salivated by the smallest quantity of this metal, while others resist the influence even of the largest quantities. In some, febrile action; in others, diarrhœa and exhaustion take place, even from moderate doses. Hence it is, that every prudent physician, if unacquainted with the previous history of his patient, makes it a special subject of inquiry to ascertain whether he has ever taken mercury previously, and how it affects him.

---

* Transactions of the Medical and Physical Society of Calcutta. vol. 1, p. 211

† Observations on the Nature and Cure of Dropsies. By John Blackall, M. D. p: 126.

‡ Manual of Midwifery. By Michael Ryan, M. D. p. 477.

Now, in the young infant, of course, as we cannot so well have the benefit of this information, mere uncertainty must necessarily attend its operation.

These, then, are the peculiarities attending the operation of mercury on young subjects, viz: that they are salivated with great difficulty, and that notwithstanding this, the effects of it are frequently more energetic and uncertain, than they are in the adult. And it is upon these as the basis, that I propose to make a few remarks bearing upon the practical application of it in young subjects.

1. If salivation occurs so rarely in children under a certain age, then it is evident that it can never be made a criterion by which to judge of its influence on their systems. To attempt, therefore, to produce this effect, as we do in adults, is manifestly improper. In cases where it is desirable to get the system under the full influence of the remedy, other modes must be resorted to for the purpose of judging to what extent the use of the article should be carried. Now this is by no means easy. Even in adults, where we have the benefit of salivation as a test, all practical physicians are aware how difficult it is frequently, to decide when it is proper to stop the use of the remedy. How much more so must this difficulty be increased in the young infant, where we are left without this guide. The only modes of judging, of course, are the character of the evacuations from the bowels, and the general impression made upon disease for which it is administered. Both these are evidently, however, uncertain. It is to be feared, therefore, that for the want of a more certain guide than we at present possess, the use of this remedy is, in many cases, unnecessarily protracted to the great detriment of the little patient. From all this the conclusion is obvious, that in the use of this article in the young subject much greater caution is necessary than in the adult.

2. The fact that mercury may prostrate and destroy a young child, even though it does not cause salivation, it is to be feared is not sufficiently appreciated, at least by some. We have known calomel given without weight or measure, to a young child, and the reason assigned to justified it was, that it could do no harm, because it would not salivate. Now it appears to me that no opinion can be more unfounded, and no practice more mischievous. Although a single dose of calomel, even though large, may be well borne by children of ordinary strength of constitution, yet even this is not entirely safe in all cases. And when these doses are frequently repeated, particularly in delicate habits, the most serious consequences may result.

3. The use of mercury in young subjects as an alterative, should in all cases be conducted with great caution. There is no practice more common than that of continuing the use of this agent in small doses, for a considerable time, and certainly none which is more liable to abuse. Under the idea that the dose is so small and from no salivation appearing, we are apt to infer that even if the medicine is not doing any good, it is certainly not doing any harm. Any improvement too, which occurs during the use of the article, is sure to be attributed to the silent operation of it on the system. Now although this is not unfrequently the case, yet it is not invariably so; and every observing physician must have been aware of cases, in which, in this way, the article has been unnecessarily and injuriously continued. In bowel complaints, under the idea of altering the secretions, it has frequently, no doubt, helped to keep up the very intestinal irritation which it was given to correct. In other cases it has developed the latent tendency to other diseases, such as Scrofula, Phthisis Pulmonalis, etc. In adults we know this to be very often the case. How much more likely is all this to happen in the young infant.

4. In the use of mercury in young children, great care should be exercised in ascertaining, as far as possible, their constitutional peculiarities. This, of course, is not in all cases easily to be done. A good deal, however, may be learned from an acquaintance with the tendencies of the parents. Wherever the parents show indications of scrofula, or where there is an hereditary predisposition to consumption, great caution ought to be exercised in the use of mercury in their offspring.

5. Mercury should be administered with great caution, in cases where a child has been sick for a considerable length of time, and when the strength of the child has been very much reduced. In this state of constitutional depression, a single cathartic dose of calomel sometimes proves fatal. We think we have seen more than one case, in which a child has been irretrievably prostrated under these circumstances, under the false impression that calomel is an innocent purgative to a child.

6. The too common practice of giving calomel as an ordinary purge, on all occasions, is certainly unjustifiable. From the facility with which it may be given, it is unquestionably resorted to in a great number of cases, where it is certainly unnecessary, and in a great number where it positively does harm. The misfortune is, that its use is not limited to an occasional dose, but it is too often given in every slight indisposition of the child. Now, in this way, there can be no question that the use of it has laid the foundation for the ruin of the constitutions of thousands. It ought to be a rule laid down and rigidly followed, that in very young children, mercury ought never to be used as a cathartic, unless there is a special reason for resorting to it. In a great majority of cases, milder cathartics are decidedly to be preferred.

In concluding these observations, I trust it may not be supposed, that my intention has been to undervalue the importance of mercury as a remedy in the diseases of children. On the contrary, no one appreciates it more highly than myself. In many cases, nothing can supply its place, and its judicious use has been, and is, the instrument of saving multitudes of lives. Notwithstanding, however, the many cautions to the contrary, it is to be feared that its use is still too general and indiscriminate. Indeed, the amount of it which is taken by the human race in one way or other, is incalculable. What is given by regular physicians, is perhaps the smallest quantity. If the public really knew how mnch of this article is swallowed unknown to themselves, in the shape of bilious pills, worm lozenges, and the white powders of the Homœopaths, they would be amazed at their credulity in deserting their old medical advisers, because they have the boldness to give them an occasional dose, and the honesty to tell them so.—*N. Y. Annalist.*

